# Histone Deacetylase Inhibitor I3 Induces the Differentiation of Acute Myeloid Leukemia Cells with *t* (8; 21) or MLL Gene Translocation and Leukemic Stem-Like Cells

**DOI:** 10.1155/2022/3345536

**Published:** 2022-08-28

**Authors:** Mengjie Zhao, Yu Duan, Jiangyun Wang, Yong Liu, Yao Zhao, Haihua Wang, Lei Zhang, Zhe-Sheng Chen, Zhenbo Hu, Liuya Wei

**Affiliations:** ^1^Laboratory for Stem Cell and Regenerative Medicine, Affiliated Hospital of Weifang Medical University, Weifang, China; ^2^School of Pharmacy, Weifang Medical University, Weifang, China; ^3^Department of Pharmaceutical Sciences, College of Pharmacy and Health Sciences, St. John's University, New York, NY, USA

## Abstract

Acute myeloid leukemia (AML) is a heterogeneous disorder characterized by the clonal expansion and differentiation arrest of leukemic cells in peripheral blood and bone marrow. Though the treatment using cytarabine-based protocol for AML patients with *t* (8; 21) translocation has improved the 5-year overall survival rate, drug resistance continues to be the principal limiting factor for the cure of the disease. In addition, very few AML patients with mixed lineage leukemia gene rearrangements (MLLr) have a desirable outcome. This study evaluated the cell differentiation effect of a potent HDAC (histone deacetylase) inhibitor, I3, and its possible mechanism on the AML cells with *t* (8; 21) translocation or MLLr and leukemic stem-like cells (Kasumi-1, KG-1, MOLM-13, and THP-1). I3 exhibited efficient anti-proliferative activity on these cells via promoting cell differentiation, accompanied by the cell cycle exit at *G*0/*G*1. Importantly, I3 showed the properties of HDAC inhibition, as assessed by the acetylation of histones *H*3 and *H*4, which resulted in blocking the activation of the VEGF (vascular endothelial growth factor)-MAPK (mitogen-activated protein kinase) signaling pathway in the Kasumi-1 cell line. These data demonstrate that I3 could be a potent chromatin-remodeling agent to surmount the differentiation block in AML patients, including those with *t* (8; 21) translocation or MLLr, and could be a potent and selective agent for AML treatment.

## 1. Introduction

Leukemia is a malignant clonal stem cell neoplasm in which immature hematopoietic cells are characterized by the failure of differentiation and unrestricted rapid proliferation [1]. Acute myeloid leukemia (AML) is an aggressive and lethal blood cancer originating from a differentiation block at the level of immature progenitors [2, 3]. Fortunately, acute promyelocytic leukemia (APL), the M3 subtype of AML, is a classic example of a disease that has become treatable with the differentiation inducer, all-trans retinoic acid (ATRA). ATRA has transformed APL from a highly fatal to highly curable disease [4, 5]. The *t* (8; 21) translocation is considered the most common structural chromosomal aberrations in patients with AML, and creates the RUNX1-RUNX1T1 fusion oncoprotein [6]. The *t* (8; 21) translocation is observed in about 40–60% of AML patients. Though the cytarabine-based protocol for AML with *t* (8; 21) has improved the 5-year overall survival rate, continuous chemotherapy may enhance toxicity and is detrimental to the patient's health [7]. Moreover, resistance to chemotherapy is a significant challenge [8]. In addition, the mixed lineage leukemia (MLL) gene rearrangements (MLLr) occur in about 10% of AML, often detected in subtypes M4 and M5, which is one of the most aggressive subtypes [9]. AML with MLLr is associated with a poor response to therapy and very few patients have either a good or an intermediate outcome [10]. Therefore, there is an urgent need to develop new therapeutics for AML with t (8; 21) translocation and the MLLr-AML. Though differentiation therapy with ATRA in APL achieves excellent success, ATRA is ineffective in treating the other subtypes of AML cases.

Histone deacetylases (HDACs) belong to an epigenetic modification enzyme family whose enzymatic activity controls the acetylation state of histones and other proteins. It can regulate chromatin accessibility and the expression of target genes [11]. It has been well established that HDACs are frequently overexpressed in human leukemia cases, which results in the abnormal regulation of chromatin remodeling and the overexpression of tumor-driven genes [12, 13]. Decades of research has shown that the inhibition of HDACs leads to cell cycle arrest, cell differentiation, or cell apoptosis, and decreases cell proliferation by altering the acetylation status of histone or non-histone proteins [13, 14]. The US Food and Drug Administration (FDA) has approved only four anti-cancer drugs targeting HDAC: SAHA (suberoylanilide hydroxamic acid), PXD-101, FK-228, and LBH-589 [13]. SAHA, a well-studied and the most famous HDAC inhibitor (HDACi), was first approved by the FDA for cancer treatment. However, it is restricted to the treatment of cutaneous T cell lymphoma [15].

I3 (Chemical name: 4-(4-(1-Ethyl-1H-indol-3-yl)butanamido)-N-hydroxybenzamide, C_21_H_23_N_3_O_3_), a HDAC inhibitor, has shown considerable potency with an inhibitory rate of 78% compared with SAHA's 59% at the concentration of 1 *μ*M [16]. Moreover, it was shown that I3 also exhibited higher inhibitory potency than SAHA against U937, U266, and HepG2 cells. In addition, I3 is a derivative of I1 with the NH in the indole ring substiuted by an ethyl group, which exhibited the activity to overcome the differentiation block in acute leukemia cells with mixed lineage leukemia gene rearrangements [17]. In the present study, we assessed whether I3 exhibited an inhibitory activity by inducing cell differentiation against AML cells with t (8; 21) translocation or MLLr and leukemic stem-like cells (Kasumi-1, MOLM-13, THP-1, and KG-1). The possible molecular mechanism of action of I3 is then explored.

## 2. Materials and Methods

### 2.1. Chemicals

**Figure 1:**
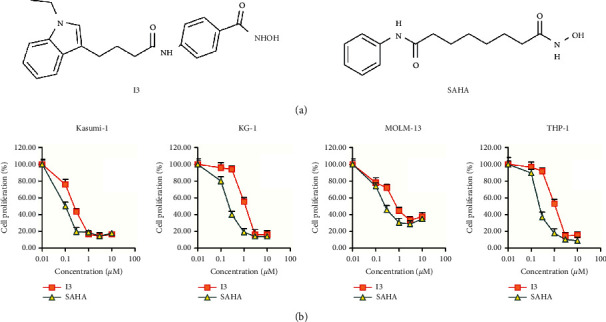
I3 inhibited the proliferation of Kasumi-1, KG-1, MOLM-13, and THP-1 cells. (a) The chemical structure of I3 and SAHA. (b) CCK-8 assay on cells treated with I3 or SAHA (0–10 *μ*M) for 72 h. Data represented as mean ± SD.

I3 was prepared by our lab. The chemical structure of I3 with a molecular weight of 366.18 and SAHA are shown in Figure 1(a). I3 or SAHA was dissolved in dimethyl sulfoxide (DMSO) to prepare a 10 mM stock solution and stored at −20°C. The desired concentrations of I3 or SAHA were obtained by diluting the stock solutions with the RPMI-1640 medium. The same concentration of DMSO as the I3 solution was used as the control. The final DMSO concentrations in the cell culture medium were not more than 0.1% and had no observable toxic effect on cells. Fluorescein Isothiocyanate (FITC)/Annexin V Apoptosis Detection Kit and Propidium iodide (PI)/RNase staining solution were obtained from BD Biosciences (San Jose, CA, USA). Cell Counting Kit-8 (CCK-8) was purchased from Solarbio (Beijing, China). PE anti-CD13 (Cat #301704 RRID: AB_314180), FITC anti-CD11b (Cat #301330 RRID: B272326), and PE anti-CD15 (cat #301906, RRID: AB_314198) were obtained from Biolegend, Inc (San Diego, CA, USA). FITC anti-CD14 (Cat #555397, RRID: 0357884) was obtained from BD Biosciences (San Jose, CA, USA). CD312 (antibodies against EMR2, Cat# MA5-28205) was obtained from Invitrogen, Inc (Carlsbad, CA, USA). MethoCult H4100 (Cat #04100) and H4435 (Cat #04435) were obtained from STEMCELL Technologies, Inc (Vancouver, BC, Canada). Antibodies against GAPDH (Cat #5174), Histone H3 (Cat #4499), Acetyl-Histone H3 (Ac-H3, Cat #8173), Histone H4 (Cat #2935), Acetyl-Histone H4 (Ac-H4, Cat #2594), RUNX1-RUNX1T1 (Cat #4336), VEGF-A (Cat #65373), MAPK/ERK (Cat #4695), and phospho- MAPK/ERK (p-MAPK/ERK, Cat #4370) were obtained from Cell Signaling Technology (Beverly, MA, USA). The Evo M-MLV RT kit (Cat #AG11706) and SYBR® Green Pro TaqHS qPCR Kit (Cat #AG11718) were purchased from Accurate Biology (Hubei, China). Spark ECL Plus A (Cat #ED0015-C), Spark ECL Plus B (Cat #ED0016-C), and RIPA buffer (Cat #EA0002) were purchased from SPARKJADE (Shandong, China).

### 2.2. Cell Lines and Cell Culture

Since there is an urgent need to develop new therapeutics for AML with *t* (8; 21) translocation and the MLLr-AML, Kasumi-1 (M2 subtype of AML with *t* (8; 21) translocation, DSMZ: ACC 220), MOLM-13 (M5 subtype of AML with MLLr, DSMZ: ACC 554), and THP-1 (M5 subtype of AML with MLLr, DSMZ No.: ACC 16) cell lines were used. In addition, KG-1 was also used to assess the effect of I3 on the proliferaton of leukemic stem-like cells, which has leukemic stem cells characteristics and was established from the bone marrow cells of an AML patient (DSMZ: ACC 14). Kasumi-1, KG-1, MOLM-13, and THP-1 cells were purchased from DSMZ (Deutsche Sammlung von Mikroorganismen und Zellkulturen GmbH, Leibniz, Germany) and maintained in RPMI-1640 medium, supplemented with 10% or 20% FBS and 1% penicillin-streptomycin, and incubated at 37°C in 5% CO_2_.

### 2.3. Cell Proliferation Assay

Kasumi-1, KG-1, MOLM-13, and THP-1 cells were seeded into 96-well culture plates at a density of about 5 × 10^3^ cells/well in 180 *μ*L medium for 24 h and were treated with 20 *μ*L of I3 at different concentrations (0.01–10 *μ*M). 10 *μ*L CCK-8 reagent was added into each well after 72 h. Then, the cells were incubated at 37°C for 4 h. Subsequently, the absorbance of each well was detected at 450 nm using an Opsys microplate reader (Dynex Technologies, Chantilly, VA, USA).

### 2.4. Cell Apoptosis Assay

Kasumi-1, KG-1, MOLM-13, and THP-1 cells were treated with I3 (0–2.4 *μ*M) or the indicated concentration of SAHA (0–1.2 *μ*M) for 72 h. First, the cells were collected and resuspended in a 1 × binding buffer. Next, the cells were mixed with Annexin V-FITC and PI for 30 min at room temperature in the dark. Finally, Beckman Coulter DxFLEX flow cytometer was used to detect the cell apoptotic rate quantitatively.

### 2.5. Cell Morphology Analysis

Kasumi-1, KG-1, MOLM-13, and THP-1 cells were cultured with a specified concentration of I3 for 72 h. Slides were made from the collected cells by cytospin, air-dried, stained with Wright-Giemsa for about 20 min, and observed for morphological features using light microscopy.

### 2.6. Analysis of Cell Surface Antigens

Kasumi-1, KG-1, MOLM-13, and THP-1 cells were treated with the indicated concentration of I3. After 72 h, the cells were centrifuged, collected, washed, and stained with monoclonal antibodies for 30 min at room temperature in the dark. The cells conjugated with the antibodies were determined with a Beckman Coulter DxFLEX flow cytometer.

### 2.7. Cell Cycle Analysis

Kasumi-1, KG-1, MOLM-13, and THP-1 cells were treated with the indicated concentration of I3 for 24, 48, or 72 h. The cells were then collected and fixed with 70% ethanol at −20°C for at least 24 h and subsequently stained with PI (50 mg/mL) and RNase A (100 mg/mL) for 30 min at room temperature in the dark. Finally, the percentage of cells in the sub-*G*1, *G*0/*G*1, *S*, and *G*2/*M* phases was detected by a Beckman Coulter DxFLEX flow cytometer (Florida, Miami, USA). ModFit software was used to analyze the percentage of cells in various phases.

### 2.8. Colony Formation Assay

Kasumi-1, KG-1, MOLM-13, and THP-1 cells were treated with I3 (0–2 *μ*M) in 2.6% methylcellulose medium containing 10% FBS and placed in a 24-wellflat-bottomed plate for two weeks. Colonies containing over 50 cells were visualized and counted using an inverted microscope (Olympus, Shinjuku-ku, Tokyo, Japan).

### 2.9. mRNA-Sequencing Analysis

Since the *t* (8; 21) translocation occurs in primary cases of AML, mRNA-Sequencing (mRNA-seq) was performed for Kasumi-1 cells. Similar to our previous work [18], cells treated with I3 for 48 h were collected for RNA extraction. Sequencing libraries were prepared, the mRNAs levels were estimated, and differential expression analysis was performed using DESeq *R* packages. The threshold of the differential expression of genes (DEGs) was set as a corrected *p*-value of 0.05 and an absolute value of log_2_FC (fold change) ≥0.58. The involved signaling pathways associated with the I3 treatment were analyzed using Gene Set Variation Analysis (GSVA). The cluster profile package of *R* software was used to conduct the enrichment analysis of DEGs. The pathways with a *p*-value of <0.05 were considered significantly enriched.

### 2.10. Real-Time PCR Analysis

According to the manufacturer's instructions, total RNA was extracted from the samples using Trizol reagent (Invitrogen Life Technologies, Carlsbad, CA, USA). The cDNA was synthesized using the Evo M-MLV RT kit (AG11706, Accurate Biology, Hunan, China). Quantitative analysis of the mRNA expression was evaluated by qPCR using S-YBR® Green Pro TaqHS qPCR Kit (AG11718; Accurate Biology), and GAPDH was used as an endogenous control. The sequences of the qPCR primers were as follow: forward GAPDH: 5′-GGCGCTGAGTACGTCGTGGAGTCCA-3′ and reverse GAPDH: 5′-AAAGTTGTCATGGATGACCTTGG-3′; forward VEGF-A: 5′-CTCACCAAGGCCAGCACATAGG-3′ and reverse VEGF-A: 5′-ATCTGGTTCCGAAAACCCTGAG-3′; forward EMR2: 5′-AGAAGCAAGTAGACAGGAGTGT-3′ and reverse EMR2: 5′-TTCTGTGCCTGATTCCAGTCG-3′; forward RUNX1-RUNX1T1: 5′-CACCTACCACAGAGCCATCAAA-3′ and reverse RUNX1-RUNX1T1: 5′-ATCCACAGGTGAAGTCTGGCATT-3′; and forward ERK: 5′-TACACCAACCTCTCGTACATCG-3′ and reverse ERK: 5′-CATGTCTGAAGCGCAGTAAGATT-3′.

### 2.11. Western Blotting Analysis

After treatment with the indicated concentration of I3 for 72 h, Kasumi-1 cells were lysed with RIPA buffer containing proteinase inhibitors. The protein lysates were separated using sodium dodecyl sulfate polyacrylamide gel (SDS-PAGE), and then transferred to the polyvinylidene fluoride (PVDF) membrane. The PVDF membrane was blocked with 10% skim milk, subsequently incubated with primary antibodies overnight at 4°C, and incubated with the appropriate secondary antibodies. The expression of proteins was visualized via the enhanced chemiluminescence (ECL) reagent detection system (Amersham Imager 600; GE Healthcare Biosciences, Pittsburgh, PA, USA).

### 2.12. Statistical Analysis

All experiments were repeated three times. Data were presented as mean ± standard deviation (SD). The comparison of each experiment group with the control was analyzed by the one-way ANOVA method using the SPSS software, and *p* < 0.05 or *p* < 0.01 was considered statistically significant.

## 3. Results

### 3.1. I3 Significantly Inhibits the Proliferation of AML Cells with *t* (8; 21) or MLLr and Leukemic Stem-Like Cells

The anti-proliferation effect of I3 on AML cells compared with SAHA was determined by the CCK-8 assay. As shown in Figure 1(b), I3 markedly inhibited the proliferation of Kasumi-1, KG-1, MOLM-13, and THP-1 cells with IC_50_ values of 0.26, 1.18, 0.86, and 1.16 *μ*M, respectively, which were comparable with that of SAHA (0.10, 0.21, 0.27, and 0.25 *μ*M, respectively). These results indicate that I3 can effectively inhibit the proliferation of AML cells with *t* (8; 21) translocation or MLL gene rearrangements and leukemic stem-like cells. Moreover, I3 had a similar anti-proliferation potency as SAHA on these cells.

### 3.2. I3 Induces Less Apoptosis in AML Cells with *t* (8; 21) or MLLr and Leukemic Stem-Like Cells

**Figure 2:**
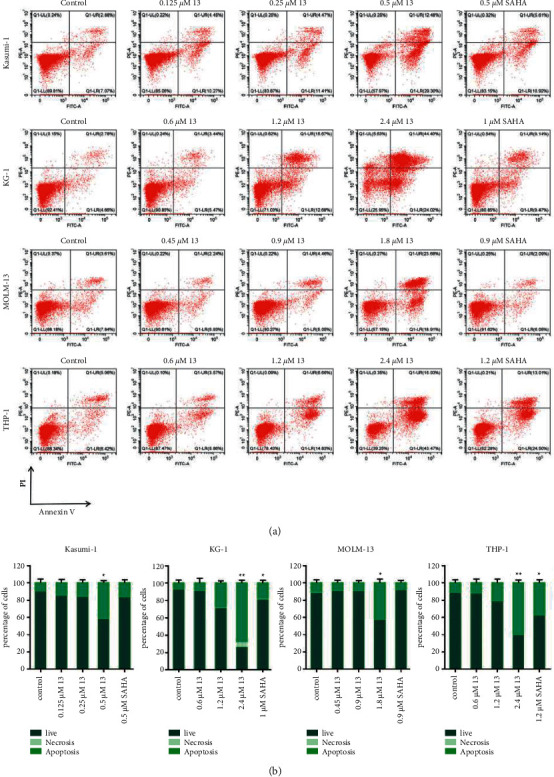
I3 induced minimal apoptosis in Kasumi-1, KG-1, MOLM-13, and THP-1 cells. (a) Cells were treated with I3 or SAHA for 72 h and cell apoptosis was determined by flow cytometric analysis. (b) Graph bars show the percentage of living cells and cells undergoing necrosis/apoptosis. Kasumi-1 cells were treated with 0.125, 0.25, and 0.5 *μ*M of I3 or 0.5 *μ*M of SAHA. KG-1 cells were treated with 0.6, 1.2, and 2.4 *μ*M of I3 or 1 *μ*M of SAHA. MOLM-13 cells were incubated with 0.45, 0.9, and 1.8 *μ*M of I3 or 0.9 *μ*M of SAHA. THP-1 cells were treated with 0.6, 1.2, and 2.4 *μ*M of I3 or 1.2 *μ*M SAHA (^*∗*^*p* < 0.05, ^*∗∗*^*p* < 0.01).

Next, we determined the apoptosis rate of cells to confirm whether the anti-proliferative effect of I3 on Kasumi-1, KG-1, MOLM-13, and THP-1 cells is associated with the induction of apoptosis. As shown in Figures 2(a) and 2(b), minimal signs of apoptosis were observed when these cells were incubated with I3 at concentrations of 0.25, 1.2, 0.9, or 1.2 *μ*M, respectively. These results indicate that the cell cycle arrest in all cells induced by I3 was not due to apoptosis. Hence, these concentrations of I3 were used in further experiments to explore their effect on cell differentiation.

### 3.3. I3 Promotes Cell Differentiation in AML Cells with *t* (8; 21) or MLLr and Leukemic Stem-Like Cells

**Figure 3:**
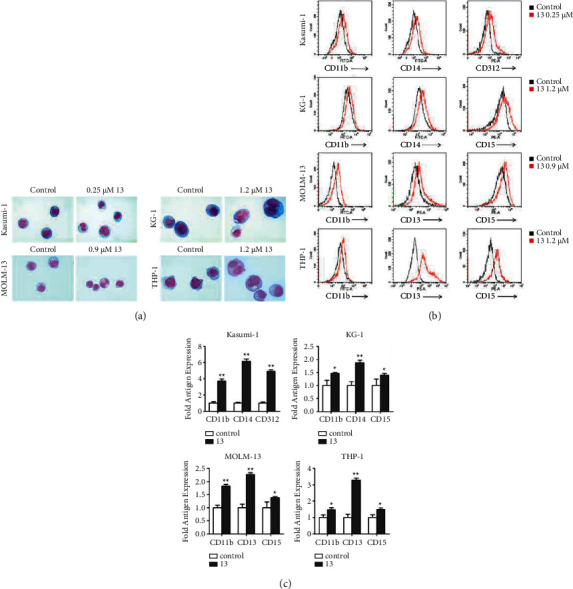
I3 induces the differentiation of Kasumi-1, KG-1, MOLM-13, and THP-1 cells. (a) The morphology of Wright-Giemsa-stained cells captured by oil immersion lens (×1,000). (b) The expression of antigens of cells measured by flow cytometry. (c) Graph bars present the mean fluorescence intensity (MFI) of antigens. Kasumi-1, KG-1, MOLM-13, and THP-1 cells were incubated with 0.25, 1.2, 0.9, or 1.2 *μ*M of I3 for 72 h (^*∗*^*p* < 0.05, ^*∗∗*^*p* < 0.01).

As I3 induces minimal apoptosis in Kasumi-1, KG-1, MOLM-13, and THP-1 cells at the indicated concentrations, we performed cell morphology analysis and cell surface antigen test to evaluate the effect of I3 on the differentiation in these cells. The cell phenotype was analyzed by determining the cell differentiation biomarkers CD11b (a granulocyte/monocyte marker), CD13 (a monocyte/macrophage marker), CD14 (a monocyte/macrophage), CD15 (a monocyte/macrophage marker), and CD321 (antibodies against EMR2, highly expressed by monocyte/macrophages [19]). As shown in Figure 3(a), after being treated with I3 for 72 h, all cells showed decreased nuclear/cytoplasmic ratio and increased cell size, revealing that I3 induced cell differentiation associated with morphological changes. Moreover, it can be seen that I3 significantly upregulated the expression of the differentiation markers CD11b, CD14, and CD312 in Kasumi-1 cells. Similarly, CD11b, CD14, and D15 were upregulated in KG-1 cells. Additionally, CD11b, CD13, and CD15 were upregulated in MOLM-13 cells and THP-1 cells (Figures 3(b) and 3(c)). Based on these results, it can be inferred that I3 could induce cell differentiation of AML cells with *t* (8; 21) or MLLr and leukemic stem-like cells.

### 3.4. I3 Induces Cell Cycle Arrest at *G*0/*G*1 in AML Cells with *t* (8; 21) or MLLr and Leukemic Stem-Like Cells

**Figure 4:**
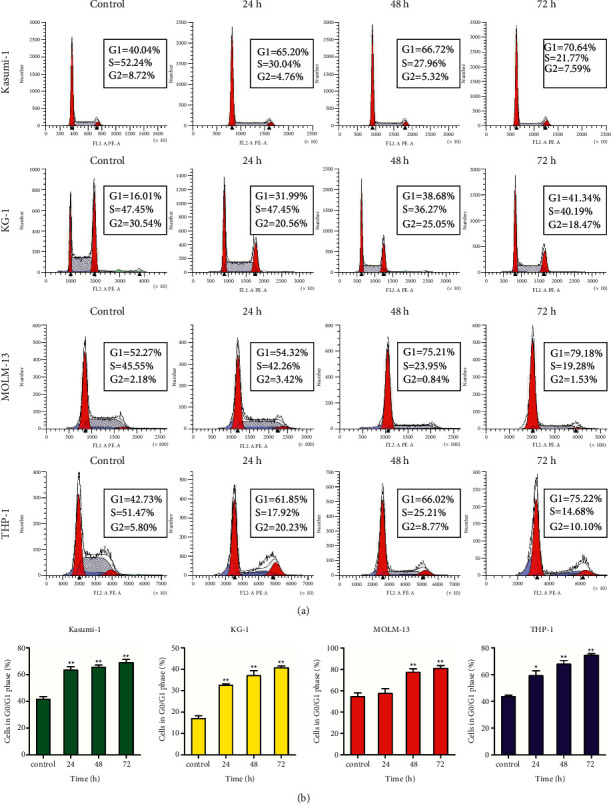
I3 induces *G*0/*G*1 cell cycle exit in Kasumi-1, KG-1, MOLM-13, and THP-1 cells. (a) Cells were treated with 0.25, 1.2, 0.9, or 1.2 *μ*M of I3, respectively, for 24, 48, or 72 h and detected by flow cytometry. (b) Graph bars show the percentage of cells in the *G*0/*G*1 phase (^*∗*^*p* < 0.05, ^*∗∗*^*p* < 0.01).

The effect of I3 on the cell cycle distribution of Kasumi-1, KG-1, MOLM-13, and THP-1 cells was assessed. It was found that the percentage of cells in the *G*0/*G*1 phase significantly increased within 72 h after I3 treatment in these cell lines (Figures 4(a) and 4(b)). These findings reveal that I3 repressed cell proliferation by inducing a cell cycle arrest at *G*0/*G*1 in AML cells with *t* (8; 21) translocation or MLLr and leukemic stem-like cells at a low concentration.

### 3.5. I3 Inhibits Colony Formation of AML Cells with *t* (8; 21) or MLLr and Leukemic Stem-Like Cells

**Figure 5:**
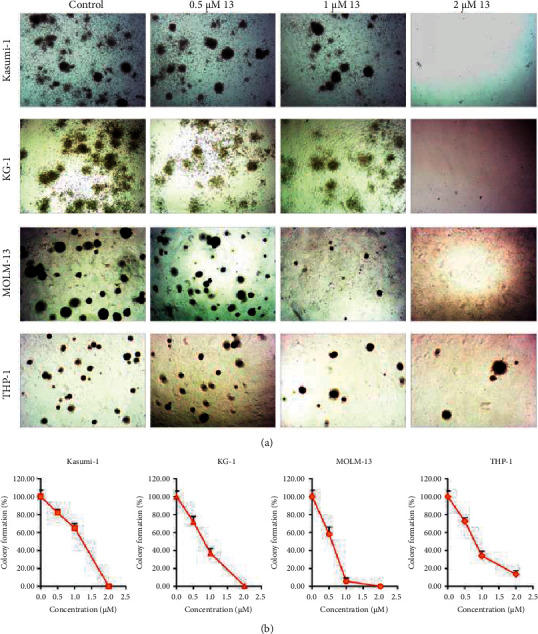
I3 suppresses colony formation in Kasumi-1, KG-1, MOLM-13, and THP-1 cells. (a) Cells were treated with I3 at concentrations of 0.5–2 *μ*M for 14 days, and the cell morphology was observed under light microscopy. (b) The effect of different concentrations of I3 on colony formation.

The effect of I3 on the colony-forming capacity of Kasumi-1, KG-1, MOLM-13, and THP-1 cells was investigated next. It was found that the number of colonies was decreased significantly with an increasing concentration of I3 (Figure 5). Moreover, Kasumi-1, KG-1, MOLM-13, and THP-1 cells rarely formed colonies when incubated with I3 at 2 *μ*M, suggesting that the differentiated cells have lost their capability to form colonies. These results indicate that I3 could significantly inhibit the colony-formation ability of AML cells with *t* (8; 21) translocation or MLLr and leukemic stem-like cells at a low concentration.

### 3.6. I3 Induces Cell Differentiation Related to the VEGF Signaling Pathway in Kasumi-1 Cells

**Figure 6:**
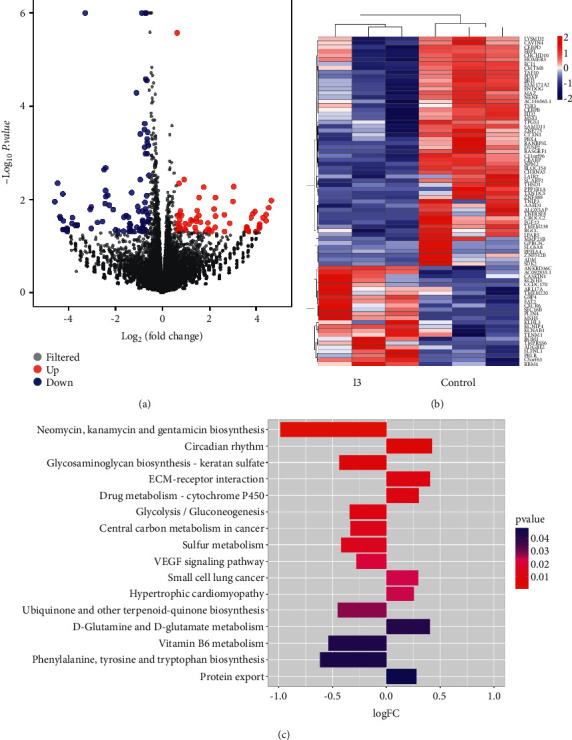
I3 promotes cell differentiation via the downregulation of the VEGF signaling pathway. (a) Volcano plots of Kasumi-1 cells. (b) The heatmap of DEGs. The heatmap bars from blue to red represent the expression levels of DEGs from low to high. (c) The significantly enriched pathways. Kasumi-1 cells were incubated with 0.25 *μ*M of I3 for 72 h.

To explore the molecular mechanism of cell differentiation mediated by I3, we performed the differential gene expression analyses using mRNA-seq in Kasumi-1 cells. We found that 86 genes were downregulated and 67 genes were upregulated, demonstrating the different effects of I3 on gene expression. The volcano plot of cells is shown in Figure 6(a). The result suggests that the mRNA expression of genes is not universally affected by I3. As shown in Figure 6(b), VEGF-A, FGF-22, and CEBP*β* were markedly downregulated, whereas ADGRE2 (EMR2) was significantly upregulated in Kasumi-1 cells incubated with I3. GSVA showed that the most optimal pathway related to cell differentiation was the VEGF signaling pathway in Kasumi-1 cells incubated with I3 (Figure 6(c)).

**Figure 7:**
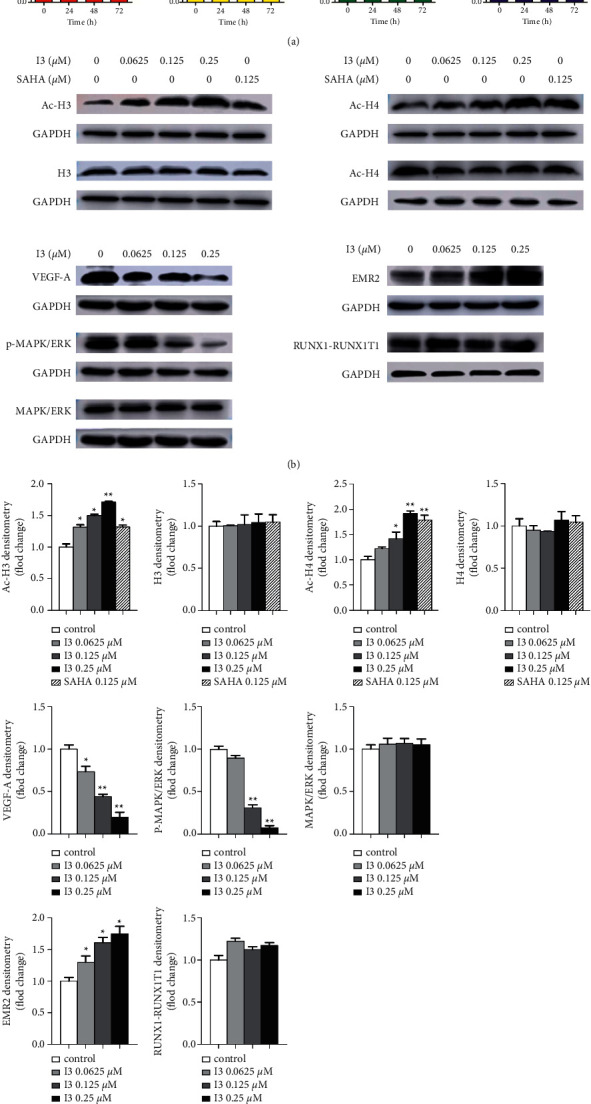
The RT-PCR and western blotting analysis of cell differentiation-related genes and proteins in Kasumi-1 cells. (a) The effect of I3 on the mRNA expression of RUNX1-RUNX1T1, ERK, VEGF-A, and EMR2 measured by RT-PCR. (b) The effect of I3 on the protein expression of H3, Ac-H3, H4, Ac-H4, VEGF-A, MAPK/ERK, p- MAPK/ERK, RUNX1-RUNX1T1, and EMR2 measured by western blotting analysis. (c) Graph bars show the protein expression quantified by the AI600 imager. Cells were incubated with 0.25 *μ*M of I3 for 72 h (^*∗*^*p* < 0.05, ^*∗∗*^*p* < 0.01).

Furthermore, some representative DEGs identified by mRNA-seq were confirmed by RT-PCR and western blotting in Kasumi-1 cells. In addition, it was found that I3 treatment markedly changed the expression of mRNA and proteins of VEGF-A and EMR2 (Figure 7(a)). These results align with the expression screened by mRNA-seq. In addition, as I3 is a potent HDAC inhibitor, we explored the effect of I3 on the inhibition of HDAC by determining the level of acetylated histones, H3 and H4, via western blotting analysis. As shown in Figures 7(b) and 7(c), the level of acetylated histones H3 and H4 increased in a concentration-dependent manner in Kasumi-1 cells incubated with I3. Moreover, I3 reduced the phosphorylation of MAPK/ERK but not the total MAPK/ERK expression. In addition, because RUNX1-RUNX1T1 fusion oncoprotein plays essential roles in AML with *t* (8; 21) translocation, we detected RUNX1-RUNX1T1 mRNA and protein expression levels in Kasumi-1 cells. It was shown that I3 did not alter the mRNA and protein expression of RUNX1-RUNX1T1.

## 4. Discussion

AML, the most common form of acute leukemia in adults, is a hematological malignancy with recurrent and refractory characteristics. The chromosomal translocation *t* (8; 21) occurs in about 40%–60% of cases of AML [20]. Clinically, the cytosine arabinoside (Ara-C)-based chemotherapy is the standard therapy for AML with *t* (8; 21) translocation. However, continuous chemotherapy may cause toxicities and can result in the development of drug resistance [21, 22]. In addition, AML with MLLr occurs in about 10% of AML and is often associated with a poor prognosis and limited response to conventional therapies [23]. Therefore, developing novel treatment compounds for newly identified targets is imperative.

HDACs catalyze the removal of the acetyl group from the *ε*-amino groups in histone or non-histone proteins, lysine residues. Acetylation and deacetylation play an essential role in the expression of genes [24]. In leukemic cells, HDACs are often overexpressed, which results in transcriptional repression. Furthermore, HDACi has been shown to induce differentiation, apoptosis, autophagy, or cell cycle arrest in hematopoietic cancers via the acetylation of histone or non-histone proteins [15, 25, 26].

I3, as a potent HDACi, has exhibited higher HDAC enzyme inhibitory activity and demonstrated higher anti-proliferation potential in HepG2, U937, and U266 cells compared with SAHA [16]. Our study is the first to show the differentiation-inducing activity of I3 in AML cells with *t* (8; 21) translocation or MLLr and leukemic stem-like cells. We showed that I3 significantly inhibited the cell proliferation and colony-forming ability of Kasumi-1, KG-1, MOLM-13, and THP-1 cells by inducing cell differentiation. The morphological changes confirm the cell differentiation by altering the expression of the cell surface antigens CD11b, CD13, CD14, CD15, or CD312. Moreover, cell differentiation was accompanied by *G*0/*G*1 cell cycle arrest. Mechanistically, the VEGF signaling pathway was demonstrated to be associated with cell differentiation in Kasumi-1 cells treated with I3. We also observed an increase in the acetylation of histones H3 and H4, suggesting that I3 targeted and inhibited HDAC, contributing to its anti-proliferative activities. In contrast, SAHA exhibited the anti-proliferation effect on leukemia cells by inducing cell aopotosis [27]. Furthermore, the effect of I3 on the HDAC inhibition activity was higher than SAHA. Taken together, we found that I3 induces cell differentiation and is effective against cell proliferation in AML cells with *t* (8; 21) translocation or MLLr and leukemic stem-like cells. Consistent results were also found with other HDACi such as valproic acid (VPA) and trichostatin A (TSA), both of which induced myeloid precursors committed to cell differentiation [28, 29].

It was shown that the VEGF signaling can stimulate cancer stemness and promote the growth and proliferation of tumor cells [30]. Several VEGF-targeted therapies have been developed [31]. The members of the VEGF family, including VEGF-A (also known as VEGF), VEGF-*β*, VEGF-C, VEGF-D, and placenta growth factor (PlGF), play essential roles in vascular biology [32]. VEGF overexpression has a demonstrated correlation with a lower remission rate and reduced overall survival in AML patients [31]. Therefore, the VEGF pathway, a key regulator of angiogenesis in hematologic malignancies, has led to several VEGF-targeted approaches [33].

Furthermore, it was demonstrated that VEGF could stimulate cell differentiation, survival, proliferation, and migration [34]. In this study, mRNA-seq showed that the VEGF signaling pathway was enriched. I3 treatment significantly decreased VEGF-A's mRNA and protein levels. Furthermore, it was shown that I3 exhibited marked HDAC inhibitory activity resulting in the acetylation of histones H3 and H4 in Kasumi-1 cells. Based on the literature, HDAC activity is required for chromatin remodeling at the VEGF promoter and VEGF mRNA expression [35]. Hence, we deduce that I3's role in decreased VEGF expression may be due to its HDAC inhibitory activity. Moreover, the MAPK/ERK signaling pathway is a signaling branch downstream of VEGF [36], and MAPKs have been demonstrated to play essential roles in regulating cell differentiation, survival, proliferation, and apoptosis [37]. We also found that p-MAPK/ERK protein level was significantly decreased with no apparent change in the expression of total MAPK/ERK protein level in Kasumi-1 cells treated with I3. Furthermore, as mentioned above, I3 treatment inhibited the proliferation of AML cells by inducing cell differentiation. This result was in line with the finding that the inhibition of MAPK/ERK phosphorylation accompanied cell differentiation [38]. In summary, the cell differentiation induced by I3 is likely associated with the block of the VEGFA/MAPK signaling pathway via the HDAC inhibition activity of I3 facilitated by the acetylation of histones H3 and H4.

In conclusion, our findings show that I3, an HDAC inhibitor and a chromatin-remodeling agent, has significant anti-proliferative effects on AML cells with *t* (8; 21) translocation or MLLr by inducing cell differentiation. The induction of cell differentiation by I3 may be associated with the blocking the activation of VEGFA/MAPK signaling pathway involving HDAC inhibition. Moreover, the activity of I3 on the HDAC inhibition was higher than SAHA. However, the effect of I3 on the primary AML samples should be further studied. In a word, I3 could overcome the cell differentiation block of AML cells with *t* (8; 21) translocation or MLLr. I3 may be a potential HDAC inhibitor worthy of further investigation including the molecular mechanism of cell differentiation of KG-1, MOLM-13, and THP-1 cells and the anti-proliferation activity on other AML cell lines and development to surmount the differentiation block in AML patients with *t* (8; 21) translocation or MLLr and leukemic stem-like cells.

## Data Availability

The original data used in this article will be available from the corresponding authors. The datasets presented in this study can be found in online repositories. The names of the repository/repositories and accession number(s) can be found here: GEO of NCBI, accession number GSE193969.
